# Bilaterally symmetrical congenital absence of radial artery: a case report

**DOI:** 10.1186/1471-2482-14-15

**Published:** 2014-03-21

**Authors:** Ying Zheng, Lei Shao, Jing-yuan Mao

**Affiliations:** 1 Cardiovascular Department of The First Teaching Hospital of Tianjin University of Traditional Chinese Medicine, 314 Anshan Western Road, Tianjin, Nankai District, China; 2 Intervension lab of The First Teaching Hospital of Tianjin University of Traditional Chinese Medicine, 314 Anshan Western Road, Tianjin, Nankai District, China

**Keywords:** Radial artery, Absence, Bilateral, Anterior interosseous artery, Ulnar artery

## Abstract

**Background:**

The radial artery is used for the access of coronary angiography and percutaneous coronary intervention, as well as for coronary artery bypass surgery. Variations of upper limb arteries are common, however, congenital absence of radial artery is scarce, and most cases were unilateral radial artery absence.

**Case presentation:**

During a coronary angiography of a 43-year-old man, we encountered a very rare bilateral congenital absence of the radial artery. For both arms, the radial arteries were not observed and the ulnar arteries were small in size, while anterior interosseous arteries were found to be the dominant artery. Coronary angiography and percutaneous coronary intervention were performed via the brachial artery since transradial percutaneous coronary intervention failed.

**Conclusion:**

The highlight of this case is that it could be the first case to be reported with bilateral absence of radial artery in adults.

## Background

Knowledge of arterial variations has a number of important implications for medical practice. The radial artery is used for the access of coronary angiography (CAG) and percutaneous coronary intervention (PCI), as well as for coronary artery bypass surgery. Therefore, doctors should be aware of such vascular anomalies of the upper limb.

Variations of upper limb arteries are common, and there are many such related reports [1-6]. Documentation of anatomic variant such as congenital absence of radial artery is scarce [7-12], and most cases were unilateral radial artery absence. During a coronary angiography of a 43-year-old man, we encountered a very rare congenital bilateral absence of radial arteries.

## Case presentation

A 43-year-old Chinese man, admitted into the inpatient cardiovascular ward on the 9^th^ July 2011, was diagnosed with anterior acute myocardial infarction (AMI) after more than 12 hours following the onset of symptoms. A transradial CAG via the right radial artery was planned to be performed one week later, before the PCI. Physical examination of the patient before the procedures found no functional disabilities of both arms and the hands were warm and well perfused. Strong palpable pulse of the arteries could also be felt at both wrists and Allen’s test was positive. Under the guidance of the pulse, the right distal artery was successfully punctured. However, the guidewire could not traverse through smoothly subsequently.

**Figure 1:**
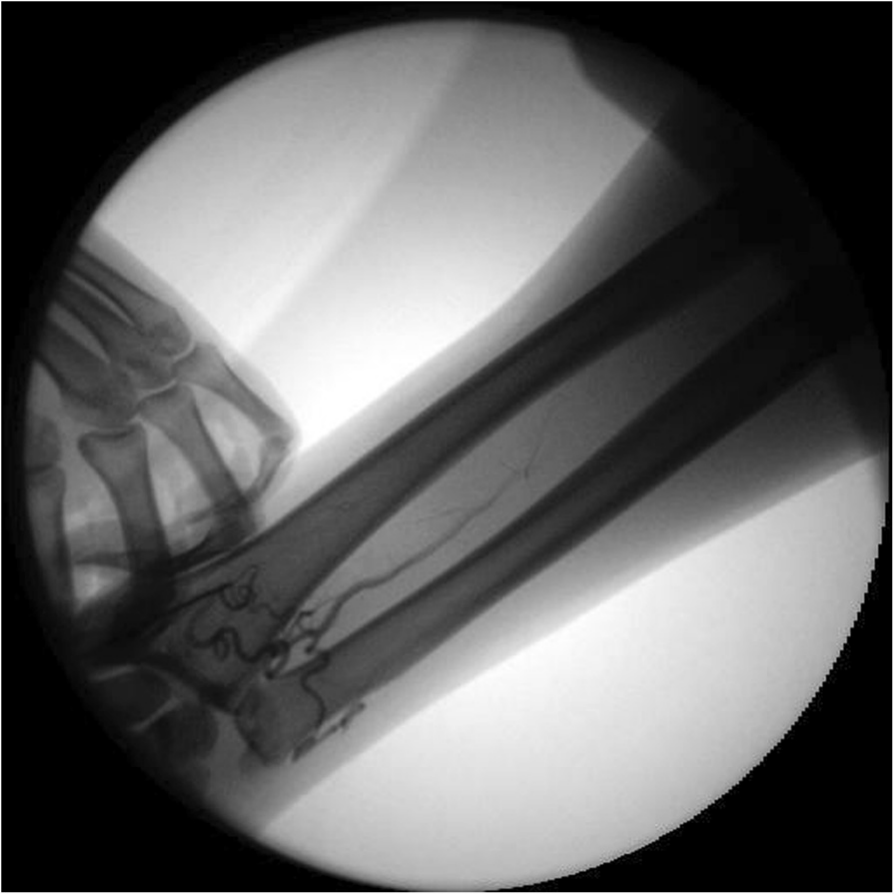
**Absence of right radial artery.** Radial artery, was not present and the anterior interosseous artery was unusually large in size. The anterior interosseous artery which coursed vertically between radius and ulna in the distal forearm branches off into two small lateral arteries to supply blood to the hands. One of the branches curled round the carpus and anastomosed with the branches of the ulnar artery, whereas the other branch traversed to radialis and became large-caliber vessel. Distal ulnar artery was small in size and together with the interosseous artery supplied blood to the hands.

**Figure 2:**
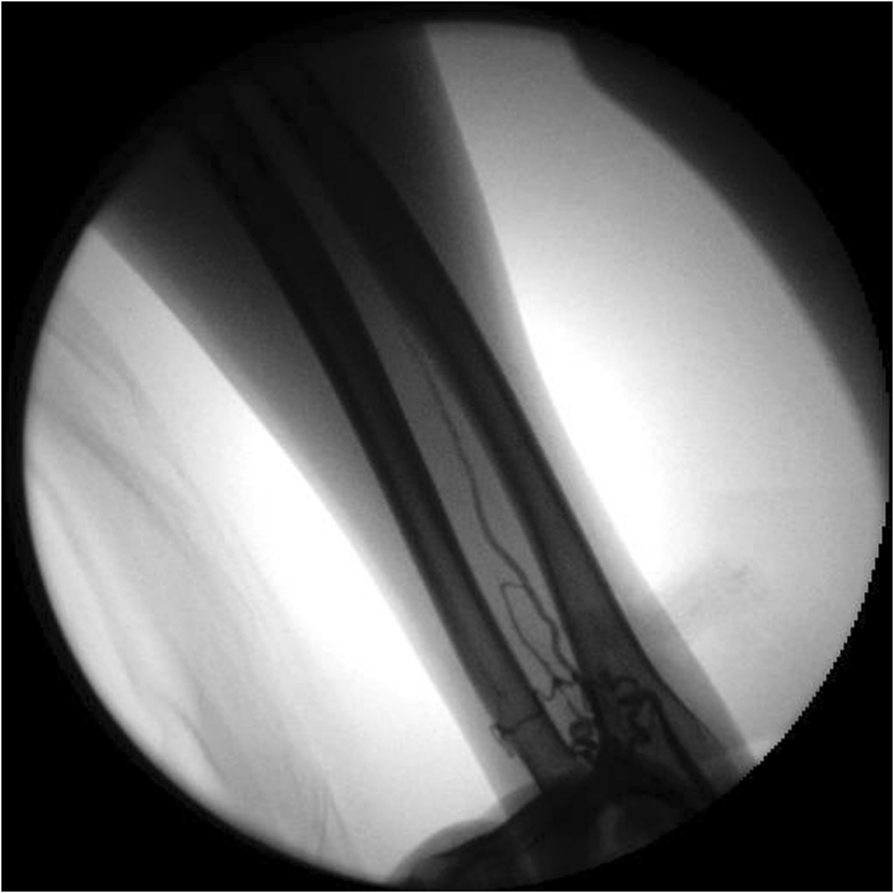
**Absence of left radial artery.** Left upper extremity arteriography revealed exactly the same pattern, the anterior interosseous artery was the dominant blood supply to the forearm and hand, distal ulnar artery was small in size, provided the part supply of the hand.

Therefore as a routine, arteriography of right upper-limb arteries was performed to delineate the anatomic features. The arteriogram of the right arm showed an absence of the radial artery (see Figure 1) and the arteriogram of the left upper limb arteries were also discovered to be similar to the right (see Figure 2). Thus, CAG and PCI were performed via the brachial artery since transradial PCI failed.

The patient had no history of operation or trauma. Right upper extremity arteriography (see Figure 1) revealed that the radial artery, arising from the brachial or axillary artery, was not present and the anterior interosseous artery was unusually large in size. The anterior interosseous artery which coursed vertically between radius and ulna in the distal forearm branches off into two small lateral arteries to supply blood to the hands. One of the branches curled round the carpus and anastomosed with the branches of the ulnar artery, whereas the other branch traversed to radialis and became large-caliber vessel. Distal ulnar artery was small in size and together with the interosseous artery supplied blood to the hands. No other vessel that coursed distally with resemblance to the radial artery was observed. An additional movie file shows this in more detail [see Additional file 1].

Left upper extremity arteriography (see Figure 2) revealed exactly the same pattern, vessels of both forearms were symmetrical in course, An additional movie file shows this in more detail [see Additional file 2].

## Discussion

Miscellaneous variations and anomalies of the arterial pattern were fairly common in upper extremities [1-6]. However, case reports were on the absence of unilateral radial artery, the congenital absence of bilateral radial arteries was rare.

In 1894, Charls [7] first reported an aged male specimen with the absence of radial artery and observed anterior interosseous artery instead in the right upper limb. However, the left radial artery was normal.

In 1966, Kadanoff and Balkansky [8] reported two cases of unilateral radial artery absence, one on the right and the other on the left.

In 1986, Poteat [9] reported the case of a Caucasian female subject with absence of left radial artery. They found that her left upper limb arterial system was developed primitively with the anterior interosseous artery as the chief blood supply to the forearm and hand. Three large terminal branches of the anterior interosseous artery supplied blood to the hand, and a small “superficial ulnar artery” perfused the hand. Radial artery or “superficial radial artery” was not observed arising from the brachial artery or the axillary. Her right side showed an unremarkable pattern of arterial distribution.

In 2001, Cowles [10] found a 44-year-old man with congenital absence of right radial artery from a right upper extremity angiography. He had a normal ulnar artery and a small interosseous artery, and his entire right hand was perfused by only the right ulnar artery. The report, however, did not mention the left upper extremity angiography.

In 2002, Suganthy [11] reported the case of a south Indian female subject with the absence of right radial artery. On the right upper limb, they observed that the brachial artery divided into the ulnar artery and large interosseous artery, whereas the radial artery was not observed. Similar to the previous report, the left extremity was not mentioned either.

In 2006, Yalcin [12] also reported a case of absence of the left radial artery. During the dissection of the right and left upper limbs of a 70-year-old female specimen, combined vascular anomalies were found. On the left arm, the absence of a radial artery with the presence of a lateral inferior superficial brachial artery and large anterior interosseous artery were observed. On the right, a trifurcation of the brachial artery was observed. It branched to the radial, ulnar and one muscular artery at the proximal one-third of the humerus.

In our case, the absence of radial arteries was bilateral and symmetrical, anterior interosseous remained as a dominant artery, while the ulnar arteries were very small in size. Both anterior interosseous artery and ulnar artery supplied blood to the forearm and hand.

## Conclusion

Bilateral absence of radial artery in adults has not been reported before, and we speculate that our case could be the first case to be reported. The radial artery is used for the access of CAG and PCI, as well as for coronary artery bypass surgery. Some examination, such as color Doppler imaging of arteries in upper extremity even arterial angiography, may be performed before cardiac catheterization or coronary artery bypass surgery.

## Consent

Written informed consent was obtained from the patient for publication of this case report and any accompanying images. A copy of the written consent is available for review by the Editor of this journal.

## Abbreviations

AMI: Acute myocardial infarction; CAG: coronary angiography; PCI: Percutaneous coronary intervention.

## Competing interests

I declare that I have no competing interests.

This manuscript is supported by Ministry of Education of People’s Republic of China “Program for Innovative Research Team in University”—Research on TCM for the Prevention and Treatment of Cardiovascular Diseases (NO.IRT1276).

## Authors’ contributions

YZ collected and analyzed the case and made substantial contributions to the writing of the manuscript. LS contributed substantially to the process of analyzing the case and writing the manuscript, and J-YM made the expert assistance in preparing the manuscript and revised it critically for important intellectual content. All authors read and approved the final manuscript.

## Pre-publication history

The pre-publication history for this paper can be accessed here:

http://www.biomedcentral.com/1471-2482/14/15/prepub

## Supplementary Material

Additional file 1 **Absence of right radial artery.** Right upper extremity arteriography revealed that radial artery was not present and the anterior interosseous artery which coursed vertically between radius and ulna was unusually large in size. Distal ulnar artery was small in size and together with the interosseous artery supplied blood to the hands. Click here for file

Additional file 2 **Absence of left radial artery.** Left upper extremity arteriography revealed exactly the same pattern, the left artery, was not present, the anterior interosseous artery was the dominant blood supply to the forearm and hand, distal ulnar artery was small in size, provided the part supply of the hand. Click here for file
